# *Camellia sinensis* L. Extract and Its Potential Beneficial Effects in Antioxidant, Anti-Inflammatory, Anti-Hepatotoxic, and Anti-Tyrosinase Activities

**DOI:** 10.3390/molecules22030401

**Published:** 2017-03-04

**Authors:** Surached Thitimuta, Pimolpan Pithayanukul, Saruth Nithitanakool, Rapepol Bavovada, Jiraporn Leanpolchareanchai, Patchreenart Saparpakorn

**Affiliations:** 1 Department of Pharmacy, Faculty of Pharmacy, Mahidol University, Bangkok 10400, Thailand; surached15@yahoo.com (S.T.); saruth_pipe@yahoo.com (S.N.); jiraporn.lea@mahidol.ac.th (J.L.); 2 Department of Pharmacognosy and Pharmaceutical Botany, Faculty of Pharmaceutical Sciences, Chulalongkorn University, Bangkok 10330, Thailand; rapepol1@hotmail.com; 3 Department of Chemistry, Faculty of Science, Kasetsart University, Bangkok 10900, Thailand; fscipnsk@ku.ac.th

**Keywords:** *Camellia sinensis* L., antioxidant, anti-inflammatory, anti-hepatotoxic, anti-tyrosinase, molecular modelling

## Abstract

The aims of this study were to investigate the potential benefits of antioxidant, anti-inflammatory, anti-hepatotoxic, and anti-tyrosinase activities of a methanolic extract of fresh tea leaves (FTE) (*Camellia sinensis* L.). The antioxidant capacity was investigated using three different methods at different temperatures. The anti-inflammatory activity was studied in vitro by the inhibition of 5-lipoxygenase assay. The anti-hepatotoxic effect was investigated in CCl_4_-induced liver injury in rats. The anti-tyrosinase activities of the FTE and its principal phenolic compounds were investigated in l-3,4-dihydroxyphenylalanine (l-DOPA) oxidation by a mushroom tyrosinase. A molecular docking study was conducted to determine how the FTE’s principal catechins interact with the tyrosinase. The FTE exhibited the best shelf life at low temperatures and demonstrated concentration-dependent antioxidant, anti-inflammatory, anti-hepatotoxic, and anti-tyrosinase effects compared to positive references. Treatment of rats with the FTE at 2000 mg/kg/day for 28 consecutive days reversed CCl_4_-induced oxidative damage in hepatic tissues by lowering the levels of alanine aminotransferase by 69% and malondialdehyde by 90%. Our findings suggest that the FTE has the capacity to scavenge free radicals and can protect against oxidative stress induced by CCl_4_ intoxication. The docking results were consistent with our in vitro data, indicating the anti-tyrosinase potency of the principal catechins.

## 1. Introduction

Free radicals and reactive oxygen species (ROS), from both endogenous and exogenous sources, have been implicated in the aetiology of several degenerative diseases, including inflammation and some hepatopathies [1]. Numerous manifestations of liver damage have been proven to be associated with redox imbalance and oxidative stress [2]. Compounds that exhibit antioxidant properties, scavenging of free radicals, and inhibition of lipid peroxidation are reported to show hepatoprotective activity [3]. Natural antioxidants exhibit a wide range of pharmacological activities and have been shown to possess multifunctional pharmacological activities such as anti-inflammatory and anti-aging properties. There has been a great deal of interest in edible plants that contain antioxidants and health-promoting phytochemicals in view of their health implications. It has become evident that phenolic compounds from natural products can reduce oxidative stress by indirect antioxidant action. Various flavonoids, which are found naturally in fruits, vegetables, and some beverages, have been demonstrated to exert antioxidant effects through a number of different mechanisms. Tea (*Camellia sinensis* L.; family Theaceae), the most popular and widely cultivated beverage in Southeast Asia, has received considerable attention among scientists due to its beneficial health effects. The health benefits associated with tea consumption have been attributed in part to the antioxidant activity and free radical-scavenging ability of the most abundant tea catechins [4,5,6]. The principal catechins present in tea leaves are epigallocatechin gallate (EGCG), epigallocatechin (EGC), epicatechin gallate (ECG), gallocatechin (GC), epicatechin (EC), and catechin. The amount of these compounds, which depends on tea variety, has been regarded as a quality indicator of tea [7]. Various biological functions of green tea, including antioxidant activities [8,9,10], anti-inflammatory activities [11], anti-melanogenic effects [12], and hepatoprotection [13,14,15,16,17], have been reported.

Tyrosinase (E.C. 1.14.18.1), a multifunctional Cu-containing enzyme that is widely distributed in nature, is found in vegetables, fruits, and mushrooms and has a key role in the browning that occurs upon brushing or long-term storage [18]. In mammals, it is a rate-limiting enzyme for melanogenesis and is responsible for skin pigmentation abnormalities such as flecks and defects [19,20]. Melanogenesis is involved in the first two steps of melanin biosynthesis, which consists of the hydroxylation of l-tyrosine and the oxidation of the product of this reaction, l-3,4-dihydroxyphenylalanine (l-DOPA), to the corresponding *o*-quinone [21]. Tyrosinase has also been linked to neurodegenerative diseases through its role in oxidizing excess dopamine to produce DOPA quinones, which are highly reactive compounds that induce neuronal damage and cell death. Most melanin synthesis inhibitors inhibit melanogenesis by inhibiting tyrosinase activity [22,23,24]. The crystallographic structure of tyrosinase has been recently established [25], enabling a closer look at its three-dimensional structure and a better understanding of its mechanism of action [26].

The aims of this study were to evaluate the potential benefits of a methanolic extract from fresh tea leaves (FTE) of *Camellia sinensis* L. cultivar Oolong No. 12 in terms of anti-lipid peroxidation, free-radical scavenging, metal chelation, and anti-inflammation in vitro. The effects of different temperatures and storage durations on the anti-lipid peroxidation activity of the FTE were also investigated. Anti-hepatotoxic activity was determined against carbon tetrachloride (CCl_4_)-induced liver damage in rats. Moreover, the inhibitory effect of this plant extract on l-DOPA oxidation by mushroom tyrosinase was investigated. A molecular docking method using the Gold v3.2 program [27] was performed to elucidate the activity of the extract. Understanding how the principal catechins in the extract interact with tyrosinase may explain how tea catechins inhibit tyrosinase and melanin formation.

## 2. Results and Discussion

### 2.1. Antioxidant Activity Assay

#### 2.1.1. Determination of Lipid Peroxidation Inhibition Using the Ferric Thiocyanate (FTC) Method

**Figure 1:**
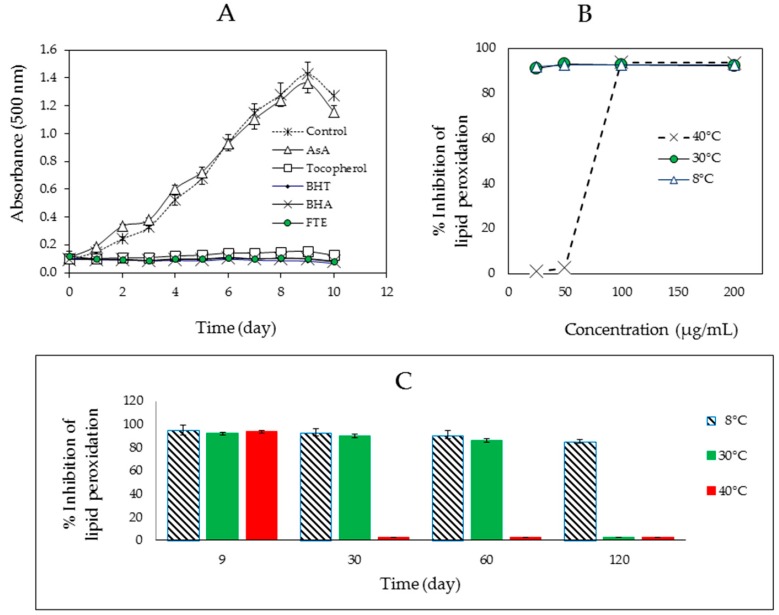
The effect of a methanolic extract of fresh tea leaves (FTE) and the positive references on the lipid peroxidation of a linoleic acid emulsion as measured by the formation of ferric thiocyanate. (**A**) Time course of linoleic acid peroxidation at 30 °C in the absence or presence of samples at a concentration of 100 µg/mL (*n* = 2); (**B**) Percent inhibition of lipid peroxidation as a function of sample concentration on day 9 of peroxidation at various temperatures (*n* = 2); (**C**) Percent inhibition of lipid peroxidation by the FTE (200 μg/mL) versus time (day) at various temperatures (*n* = 2).

At a concentration of 100 µg/mL, the FTE delayed the oxidation of linoleic acid compared to the reference standards (butylated hydroxyanisole; BHA, butylated hydroxytoluene; BHT and tocopherol) and, based on the low absorbance values observed, exhibited significantly higher activity than ascorbic acid (AsA) and the control (*p* < 0.05) at 30 °C (Figure 1A). The extract demonstrated potent inhibition of lipid peroxidation of up to 85%–95% during the storage period of 120 days at 8 °C and 60 days at 30 °C, but less than 5% inhibition at 40 °C (Figure 1B,C). These results reflect the instability of the FTE at high temperatures. This finding was not surprising because heating causes acceleration of the initial reactions that result in a decrease of the activity of the added antioxidants [28].

#### 2.1.2. Determination of Free Radical Scavenging Activity Using the 2,2-Diphenyl-1-picrylhydrazyl (DPPH) Assay

**Figure 2:**
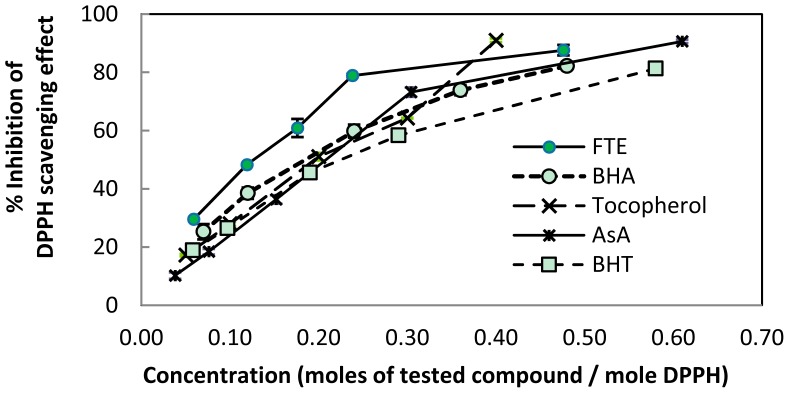
The percent inhibition of DPPH radical-scavenging activity. Data are expressed as the mean ± standard deviation (SD) (*n* = 2). The values are significantly different at *p* < 0.05. The reduction of the DPPH radical was measured by the decrease of absorbance at 515 nm as a function of the concentration of the FTE and the reference standards.

The reduction of DPPH by the FTE occurred in a concentration-dependent manner (Figure 2). The percent inhibition of DPPH was more pronounced in the presence of the FTE compared to AsA, BHA, BHT, and tocopherol, suggesting highly efficient free radical-scavenging activity. The order of potency, as determined by the 50% inhibition of the DPPH scavenging effect (SC_50_), was FTE > BHA > tocopherol > AsA > BHT (Table 1). The powerful antioxidant activity of the FTE was primarily attributed to the presence of tea catechins [29].

#### 2.1.3. Chelation of Ferrous Metal Ions

**Figure 3:**
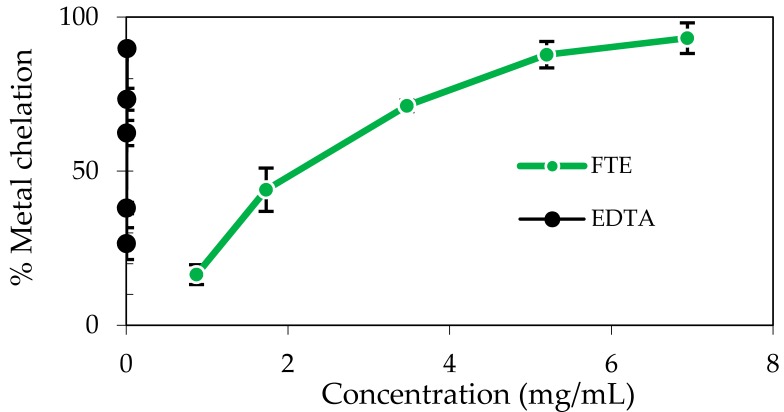
The metal chelating effect of the FTE and EDTA. Data are expressed as the mean ± SD (*n* = 2).

The FTE exhibited a metal-chelating effect in a concentration-dependent manner (Figure 3). Based on the IC_50_ in Table 2, the iron chelation capacity of the FTE (2.08 ± 0.10 mg/mL) was much lower than that of ethylenediaminetetraacetic acid (EDTA) (0.01 ± 0.00 mg/mL), which reflected the natural iron chelation capacity of the FTE and the ability of the FTE to bind iron. These factors might also contribute to its inhibition of lipid peroxidation.

### 2.2. Determination of In Vitro Anti-Inflammatory Activity by the Inhibition of 5-Lipoxygenase (5-LOX)

**Figure 4:**
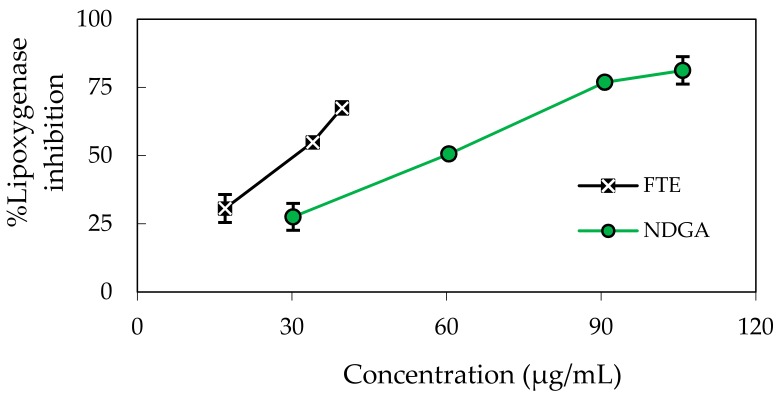
The percent inhibition of soybean lipoxygenase among varying concentrations of the FTE and the positive reference (NDGA). Data are expressed as the mean ± SD (*n* = 3).

The extract exhibited a concentration-dependent inhibition of soybean lipoxygenase with an IC_50_ of 30.60 ± 0.40 µg/mL compared to that of the reference standard (nordihydroguaiaretic acid, NDGA), with an IC_50_ of 59.26 ± 0.92 µg/mL (Figure 4, Table 2). Inflammatory processes become pathogenic due to lipoxygenase catalysing the synthesis of leukotrienes via the transfer of oxygen radicals [30]. It is possible that the termination of leukotriene biosynthesis by the FTE could be attributed to the potential abilities of the FTE to scavenge free radicals generated within the active site of the enzyme. The results of this study support earlier reports that natural products possess the ability to inhibit 5-LOX in addition to free radical-scavenging activity [31].

### 2.3. Determination of the Anti-Hepatotoxic Effects of the FTE on CCl_4_-Induced Liver Injury in Rats

The activities of the biochemical parameters in the normal control, CCl_4_, the positive control, and the treated groups of rats with CCl_4_-induced liver injury are presented in Table 3. The activities of alanine aminotransferase (ALT) and malondialdehyde (MDA) were significantly (*p* < 0.05) increased in the groups treated with CCl_4_ compared to the normal controls. Daily treatment with the FTE at a dose of 500 mg/kg produced a marginal change in ALT (3.16%) and MDA (24.44%) activities, while higher doses (1000 mg/kg and 2000 mg/kg) of the FTE produced a dose-dependent, significant decrease in serum ALT (69%) and hepatic MDA (90%) when compared with the levels in the CCl_4_ control (*p* < 0.05). The reversing effect of the group treated with silymarin towards normalization was more potent at 80% for ALT and 79% for MDA. There were no significant changes in ALT and MDA activities in healthy rats treated with the extract alone. The abnormally high serum levels of ALT (301.04 ± 13.88 U/L) and MDA (0.14 ± 0.01 nmol/g liver) observed in the CCl_4_-treated rats reflected the consequences of CCl_4_-induced liver dysfunction and indicated oxidative damage to hepatic cells compared to the normal treated group (ALT = 25.49 ± 2.41 U/L, MDA = 0.02 ± 0.00 nmol/g liver).

Taken together, the results of the FTC and DPPH assays suggest that FTE protects against lipid peroxidation by donating hydrogen atoms to terminate the radical chain reaction. The capacity of FTE to bind iron may also contribute to its inhibition of peroxidation. The FTE possess the ability to inhibit 5-LOX, as well as free radical scavenging activity. This finding implied that treatment with the FTE caused the inhibition of lipid peroxidation and thus protected against the damaging effects of free radicals induced by the administration of CCl_4_. Thus, the administration of the FTE resulted in a hepatoprotective effect against the toxic effects of CCl_4_.

### 2.4. Determination of Mushroom Tyrosinase Inhibition In Vitro

**Figure 5:**
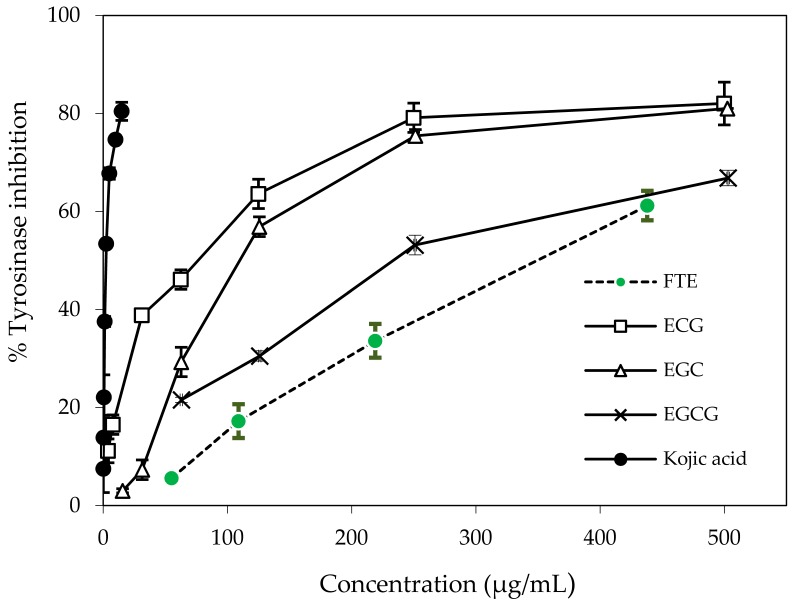
Concentration-dependent inhibition of mushroom tyrosinase by the FTE, its principal phenolic components (ECG, EGC, and EGCG) and kojic acid. Tyrosinase activity was measured using l-DOPA as the substrate. Each value represents the mean ± SEM (*n* = 2).

The inhibitory effects of the FTE and a well-known tyrosinase inhibitor, kojic acid (KA), on l-DOPA oxidation by mushroom tyrosinase were concentration-dependent, as shown in Figure 5. The order of potency as determined by the half-inhibition concentration (IC_50_, µg/mL) was KA (2.28 ± 0.03) > ECG (76.50 ± 1.50) > EGC (110.00 ± 0.00) > EGCG (234.00 ± 6.00) > FTE (349.00 ± 9.00) (Table 2).

### 2.5. Molecular Modelling

**Figure 6:**
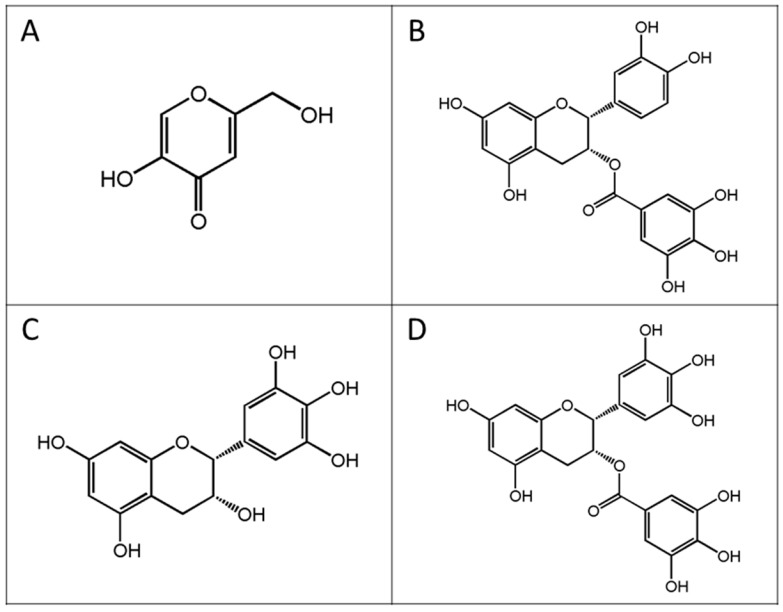
The structures of the compounds used in this study. (**A**) Kojic acid (KA); (**B**) Epicatechin-3-gallate (ECG); (**C**) Epigallocatechin (EGC); (**D**) Epigallocatechin-3-gallate (EGCG).

**Figure 7:**
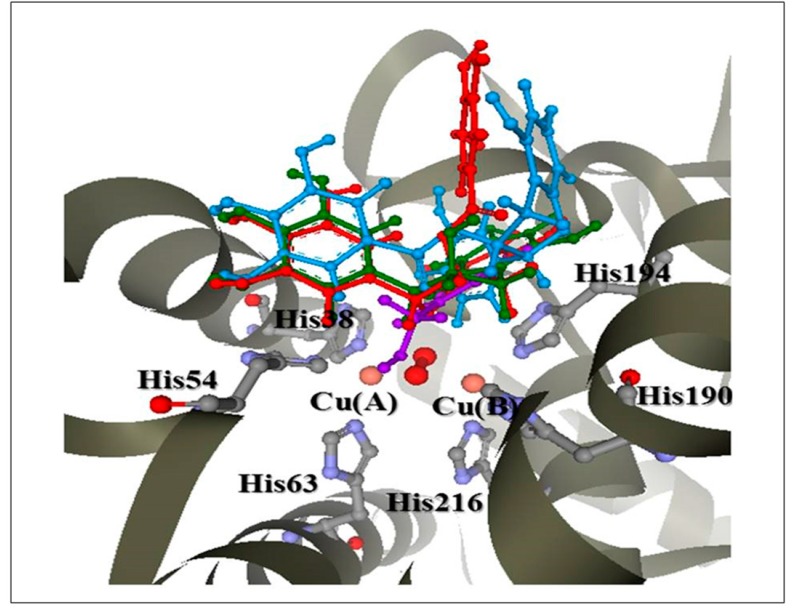
The docked conformations of the ligand structures in the tyrosinase-binding site. KA (Purple), ECG (Blue), EGC (Green), and EGCG (Red).

**Figure 8:**
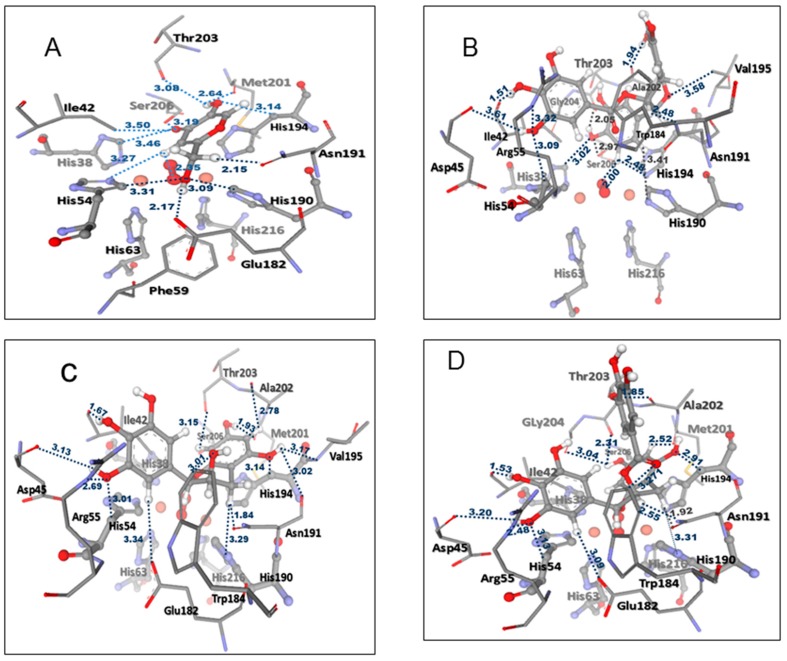
The distances (in Å) between the residues in the tyrosinase-binding pocket and ligands: (**A**) KA; (**B**) ECG; (**C**) EGC; and (**D**) EGCG. Oxygen, nitrogen, carbon, hydrogen, sulfur, and copper atoms are presented in red, violet, grey, white, yellow, and orange color, respectively.

The docking results agreed well with the observed in vitro data, in which the anti-tyrosinase activity of KA was much higher than that of the FTE and its principal phenolic compounds ECG, EGC, and EGCG. Docked KA showed more H-bonding interactions with important amino acids located close to the two copper atoms in the active site of the enzyme compared to ECG, EGC, and EGCG. The selected docked conformations of the structures of KA, ECG, EGC, and EGCG (Figure 6) in the tyrosinase structure are shown in Figure 7, and their GoldScores were 32.40, 57.46, 51.25, and 57.03, respectively. The docked conformation revealed that all ligands were located in the hydrophobic binding pocket surrounding the binuclear copper active site. The locations of docked compounds were consistent with those of docked 2,4-resorcinol derivatives, which can contribute to the potency of the observed tyrosinase inhibitory effect [32]. The docked conformation of KA revealed H-bonding to an oxygen atom of peroxide and the His38, Ile42, His54, Glu182, His190, Asn191, Met201, Thr203, and Ser206 residues in the active site (Figure 8A). Kojic acid also interacted with the His194 ring and showed specific H-bonding to the Phe59 residue. With respect to the docked conformations of ECG, EGC, and EGCG (Figure 8B–D), strong H-bonding was observed with the Ile42 residue. Moreover, an interaction with the His194 ring and common H-bonding to the Asp45, His54, Arg55, Trp184, His190, Asn191, Ala202, and Thr203 residues occurred. For the docked conformation of ECG, additional H-bonding interactions were observed with an oxygen atom of peroxide and the His38, His194, Val195, Gly204, and Ser206 residues. The docked conformation of EGC also exhibited H-bonding interactions with the Glu182, His194, Val195, Met201, and Ser206 residues. For the docked conformation of EGCG, H-bonding interactions occurred with an oxygen atom of peroxide and the His194, Met201, Gly204, and Ser206 residues. Compared to EGC and EGCG, ECG formed specific H-bonds with the His38 residue, which was also observed for docked KA. His38, His54, His190, and His194 are important amino acid residues that interact with the two copper ions in the active site. With respect to docked EGC and EGCG, EGC formed specific H-bonds with Glu182, which was also observed in docked KA. Therefore, the docking results agreed well with the results of the in vitro data, which indicated that the anti-tyrosinase potency of the principal catechins of the tea extract was in the order of ECG > EGC > EGCG.

## 3. Materials and Methods 

### 3.1. Plant Material

Freshly picked tea leaves (*Camellia sinensis* L., family Theaceae, cultivar Oolong No. 12) (FTE), including only two leaves and a bud from the terminal branches, were provided by Doi Mae Salong’s tea plantation, Chiang Rai, Thailand (5 May 2005). The identities of the samples were confirmed, and a voucher specimen (No. P.B. 20009) was deposited at the Museum of Natural Medicine, Faculty of Pharmaceutical Sciences, Chulalongkorn University, Bangkok, Thailand.

### 3.2. Chemicals

Ascorbic acid (AsA; ≥99%), 2,2-diphenyl-1-picrylhydrazyl (DPPH; ≥85%), gallic acid (GA; ≥98%), kojic acid (KA; ≥98%), and 2-thiobarbituric acid (TBA; ≥98%) were purchased from Fluka (Buchs, Switzerland). 5-Lipoxygenase (5-LOX, EC 1.13.11.34, 80,000 units/mg solid), ethylenediaminetetraacetic acid (EDTA; 99.8%), (−)-epicatechin (EC; ≥98%), (−)-epicatechin-3-gallate (ECG; ≥98%), (−)-epigallocatechin (EGC; ≥95%), (−)-epigallocatechin-3-gallate (EGCG; ≥95%), 3-(2-pyridyl)-5,6-bis(4-phenylsulfonic acid)-1,2,4-triazine (ferrozine; ≥99%), l-3,4-dihydroxyphenylalanine (l-DOPA; ≥98%), mushroom tyrosinase (EC 1.14.18.1, ≥1000 units/mg solid), nordihydroguaiaretic acid (NDGA; ≥97%), and silymarin (batch 107K0762; 47% of silybin) were obtained from Sigma Chemical Co., Ltd. (St. Louis, MO, USA). Butylated hydroxyanisole (BHA; 99.5%) and butylated hydroxytoluene (BHT; 99.5%) were obtained from Nikki-Universal Co., Ltd. (Tokyo, Japan). dl-α-tocopherol was purchased from BASF SE (Ludwigshafen, Germany). All the chemicals and solvents were of analytical grade.

### 3.3. Animals

Male Sprague-Dawley rats weighing 150–200 g were used. They were housed in groups of 2–3 rats/cage with free access to a granular standard diet (C.P. MICE FEED; S.W.T. Co., Ltd., Samut Prakan, Thailand) and drinking water. They were maintained under a 12 h light-dark cycle at a temperature of 23 ± 2 °C and a humidity of approximately 65%–70% for at least 1 week before use. The basic parameters related to the animals such as body weight, food intake, etc. were not monitored during the experiment but were only evaluated at the beginning of the experiment.

The experimental protocol (No. 0014) was approved by the Institutional Animal Care and Use Committee of the Faculty of Pharmacy, Mahidol University, in accordance with the Ethical Principles and Guidelines for the Use of Animals for Scientific Purposes by the National Research Council of Thailand, Ministry of Science and Technology.

### 3.4. Preparation and Standardization of Plant Extract

The leaves (10.66 kg) were thoroughly washed, chopped, and macerated using methanol (70%) as the extracting solvent at a ratio of fresh tea leaves/solvent = 1:2 (*w*/*v*) for 24 h at room temperature (30 °C). Subsequently, the methanolic extract was filtered and the residue was remacerated 4 times with fresh methanol (70%). The combined methanolic solution was centrifuged at 2000 rpm for 15 min in a Hettich Roto Magna^®^ and concentrated in a rotary evaporator (model SB-650, Tokyo Rikakikai Co. Ltd., Japan) at 35 °C. The extract was decaffeinated with dichloromethane, evaporated under reduced pressure, and then freeze dried to produce a crude, fresh tea leaf extract with a yield of 9.40% (*w*/*w*). The total phenolic content of the extract was analysed using the Folin-Ciocalteu colorimetric method [33]. The FTE was found to contain 245.20 ± 4.92 mg/g of total phenolic compounds expressed as the gallic acid equivalent (GAE, mg/g of extract).

The dried extract was analysed for the contents of four catechins—EC, ECG, EGC, and EGCG—using a reverse-phase high-performance liquid chromatography (HPLC) method [34]. The HPLC analysis was performed using a Shimadzu model LC-10AD (Tokyo, Japan), consisting of a binary pump, SIL-10AD*VP* autosampler, and an SPD-10A*VP* detector (UV-Vis absorbance detector) and equipped with an Alltech Altima^®^ C18 column (4.6 × 150 mm, 5 μm). An isocratic mode was used, and the mobile phase was 0.1% phosphoric acid in water/acetonitrile (85:15, *v*/*v*). The flow rate was 1 mL/min, and the injection volume was 10 μL. Detection was conducted at 280 nm, and the identification of individual catechins was based on the comparison of the retention time and UV spectrum of unknown peaks with those of authentic reference standards. Four individual catechins were quantified in the FTE by comparison with the generated standard curves (peak area vs. concentration). The methanolic extract of the freshly picked tea leaves was found to contain 11.7, 16.1, 72.1, and 89.7 mg/g dry weights of ECG, EC, EGCG, and EGC, respectively.

### 3.5. Antioxidant Activity Assay

#### 3.5.1. Determination of Lipid Peroxidation Inhibition Using the FTC Method

The FTC method [35] was used to determine the in vitro inhibition of linoleic acid peroxidation. In this study, 0.1 mL of each sample was added to the assay mixture. Antioxidant activity was calculated as the percent inhibition of linoleic acid peroxidation versus control. BHA, BHT, tocopherol, and AsA were used as positive references. The effects of temperature variation and storage period on the inhibition of lipid peroxidation were investigated by incubating the samples at 8 °C, 30 °C, and 40 °C during a period of up to 120 days.

#### 3.5.2. Determination of Radical Scavenging Activity Using the DPPH Test

The DPPH method [36] was used to determine the free radical-scavenging potential of each sample. The test sample (0.1 mL) was added to 3.9 mL of DPPH solution (6 × 10^−5^ M). The absorbance was measured at 515 nm after 30 min of the reaction at room temperature (30 °C). The antiradical activity was calculated as a percentage of DPPH decolouration versus a control. The results were expressed as the concentration of the FTE that scavenged 50% of the free radicals from the reaction mixture (SC_50_). The percent DPPH scavenging effect was calculated by using following equation [37]:

(1) $$\mathrm{DPPH}\mathrm{scavenging}\mathrm{effect}(\%)\mathrm{or}\mathrm{Percent}\mathrm{inhibition}=[\frac{\mathrm{Ao}\;-\;\mathrm{At}}{\mathrm{Ao}}]\text{×}100$$

where A_o_ is the absorbance of the control reaction and A_t_ is the absorbance in the presence of a test or standard sample. AsA, BHA, BHT, and tocopherol were used as positive references.

#### 3.5.3. Chelation of Ferrous Metal Ions

The method of Gülçin et al. [38] was followed. The reaction mixture, containing different concentrations of each sample, FeCl_2_ (2 mM), and ferrozine (1.5 mM), was adjusted to a total volume of 4 mL with absolute methanol, shaken well, and incubated for 10 min at room temperature (30 °C). The absorbance of the mixture was measured at 562 nm against a blank. EDTA was used as the positive reference. The percent inhibition of ferrozine-Fe^2+^ complex formation was calculated against a control. The results were expressed as the concentration of the FTE that chelated 50% of metal ions (IC_50_).

### 3.6. Determination of In Vitro Anti-Inflammatory Activity by the Inhibition of 5-LOX

The method of Baylac and Racine [39] was followed. The assay mixture (3 mL) consisted of 2 mM linoleic acid, 50 µL of each sample at various concentrations in distilled water, and 750 µL of freshly prepared 5-LOX (3200 units/mL) in 0.2 M borate buffer (pH 9). The reaction was initiated by the addition of linoleic acid. The increase in absorption at 234 nm was monitored for 5 min at 25 °C. The initial reaction rate was determined from the slope of the straight-line portion of the curve, and the percentage inhibition of enzyme activity was calculated by comparison with the positive control, NDGA. The extent of inhibition of the test samples was expressed as the percentage of concentration necessary to achieve 50% inhibition (IC_50_).

### 3.7. Determination of the Anti-Hepatotoxic Effects of the FTE on CCl_4_-Induced Liver Injury in Rats

Animals were divided into six groups consisting of eight animals in each group. Group 1 served as the normal control and received distilled water (1 mL/kg body weight per day). Group 2 served as the CCl_4_-treated control and received distilled water (1 mL/kg body weight per day). Groups 3–5 (extract treatment) were treated with the FTE at doses of 500, 1000, and 2000 mg/kg body weight per day, respectively. Group 6 (extract alone) was treated with the FTE at a dose of 2000 mg/kg body weight per day. Silymarin was selected as an internal positive control in this study and therefore, group 7 served as the silymarin-positive control-treated group and received a dose of 100 mg/kg body weight per day. All treatments were administered orally for 28 days. With the exception of groups 1 and 6, all other groups received CCl_4_:liquid paraffin (1:2, 1 mL/kg body weight, i.p.) on days 23, 26, and 29. The rationale to select the doses of CCl_4_ was based on the literature study with modification [40,41,42,43]. On day 30, the rats were anesthetized using diethyl ether; blood samples were collected from the abdominal artery, and the liver was removed. The serum was separated from the blood, and the serum and the liver samples were stored at −80 °C until analysis.

#### 3.7.1. ALT Assay

The activity of ALT was determined from the obtained serum and evaluated using an ALT assay kit (Audit, Ireland). One hundred microliters of the sample serum were added to 1 mL of test reagent in a 1 cm light path cuvette. After incubation at 37 °C for 1 min, measurements were taken of the changes in optical density per minute (∆OD/min) during the next 3 min at a wavelength of 340 nm. Enzyme activity was expressed as U/L and calculated as follows:

(2) Enzymeactivity(U/L)=∆OD/min×1746

#### 3.7.2. Thiobarbituric Acid (TBA) Assay

The method of Ohkawa et al. [44] was followed for this experiment. The mixture was prepared by combining 0.1 mL of liver homogenate with 0.2 mL of 8.1% sodium lauryl sulphate (SLS), 1.5 mL of 20% acetic acid solution at pH 3.5, and 1.5 mL of 0.8% aqueous solution of TBA. The mixture was brought up to 4 mL with distilled water and heated at 95 °C for 60 min in a water bath. After cooling, 1 mL of distilled water and 5 mL of a mixture of *n*-butanol and pyridine (15:1, *v*/*v*) were added, and the mixture was shaken vigorously. After centrifugation at 4000 rpm for 10 min, the absorbance of the organic layer (upper layer) was measured at 532 nm. 1,1,3,3-Tetramethoxypropane (TMP) was used as an external standard. The lipid peroxide level was expressed as nmol of MDA.

### 3.8. Determination of Mushroom Tyrosinase Inhibition In Vitro

The dopachrome method [45] was used to determine the anti-tyrosinase activity of the FTE and its principal catechins (ECG, EGC, and EGCG) compared with that of the standard tyrosinase-inhibitor control, KA. Briefly, 120 µL of 20 mM phosphate buffer (pH 6.8), 40 µL of 48 units/mL mushroom tyrosinase, and 20 µL of various sample concentrations with or without enzyme were placed in the wells of a 96-well microplate. After pre-incubation at 25 °C for 10 min, 20 µL of 0.85 mM L-DOPA was added, and the plate was subsequently incubated at 25 °C for 20 min. The amount of dopachrome was measured at 492 nm in an ELISA plate reader (Anthos 2010, Anthos Labtec Instruments GmbH, Wals, Austria). The extent of inhibition by the test samples was expressed as the percentage of concentration necessary to achieve 50% inhibition (IC_50_).

### 3.9. Molecular Modelling

Docking studies of ECG, EGC, EGCG and the positive reference (KA) were carried out. The structures of these compounds (Figure 6) were constructed and optimized at the HF/3-21G level of theory using a Gaussian 03 program [46]. For the preparation of the tyrosinase structure, the crystal structure of the oxy form of tyrosinase was taken from the Protein Data Bank (PDB code 1wx2) [25]. Caddie protein (ORF378) and water molecules were removed. Hydrogen atoms were added to the enzyme using the SYBYL version 7.2 program (TRIPOS, Association Inc., St. Louis, MO, USA). The molecular docking method was conducted using the Gold version 3.2 program [27] to study the binding orientation of ECG, EGC, EGCG, and KA in the tyrosinase structure. The radius of the binding site was set to 10 Å. The default parameters of the automatic settings were used for setting the genetic algorithm parameters. The docked conformation with the highest GoldScore was selected to analyse the mode of binding.

### 3.10. Statistical Analysis

The data were presented as the mean ± SD or mean ± SEM. All statistical analyses were carried out using SPSS 13.0 for Windows. Significant differences (*p* < 0.05) between the means were assessed using one-way ANOVA, followed by Scheffé’s method or Dunnett’s T3 test for multiple comparisons.

## 4. Conclusions

In conclusion, the FTE demonstrates potential beneficial antioxidant, anti-inflammatory, and anti-hepatotoxic activities both in vitro and in vivo. The FTE is most effective at low temperatures and degrades rapidly at high temperatures. The docking results provide an explanation for the abilities of the catechin compounds to inhibit tyrosinase. This information may be helpful to use as a guideline for designing potent tyrosinase inhibitors.

## Figures and Tables

**Table 1:** The 50% inhibition of the DPPH scavenging effect, SC_50_ (moles of tested compound/mole DPPH), induced by the FTE and the reference standards.

Tested Samples	SC_50_ (Moles of Tested Compound/Mole DPPH)
FTE	0.128 ± 0.004
Reference standards:	
BHA	0.184 ± 0.011
BHT	0.224 ± 0.005
Tocopherol	0.196 ± 0.005
AsA	0.209 ± 0.006

**Table 2:** The constituents and contents of the FTE and the concentration providing 50% inhibition of the iron(II) chelating capacity, lipoxygenase, and tyrosinase activities of the FTE and its four isolated pure compounds, including reference standards. The values represent the mean ± SD or the mean ± standard error of the mean (SEM).

FTE and Its Isolated Compounds	Contents ^a^		Iron(II)-Chelating ^b^ (IC_50_, mg/mL)	Anti-Lipoxygenase ^b^ (IC_50_, µg/mL )	Anti-Tyrosinase ^a^ (IC_50_, µg/mL )
	**% *w*/*w***	mg/g Dry Weight			
FTE ^c^, total phenolic content	24.52	245.20 ± 4.92	2.08 ± 0.10	30.60 ± 0.40	349.00 ^d^ ± 9.00
**Constituents:**					
Epicatechin (EC)	1.60	16.05± 0.25	-	-	Nd
Epicatechin-3-gallate (ECG)	1.17	11.70 ± 0.20	-	-	76.50 ^d,e^ ± 1.50
Epigallocatechin (EGC)	8.97	89.70 ± 2.00	-	-	110.00 ^d,e^ ± 0.00
Epigallocatechin-3-gallate (EGCG)	7.21	72.10 ± 2.30	-	-	234.00 ^d,e^ ± 6.00
Unidentified phenolic content	5.57	-	-	-	-
**Reference Standards:**					
Ethylenediaminetetraacetic acid (EDTA)	-		0.01 ± 0.00	-	-
Nordihydroguaiaretic acid (NDGA)	-		-	59.26 ± 0.92	-
Kojic acid (KA)	-		-	-	2.28 ± 0.03

^a^ The values are mean ± SEM. ^b^ The values are mean ± SD. ^c^ FTE concentration was calculated based on the gallic acid equivalent (GAE, mg/g of extract). ^d^ Significant difference compared to kojic acid (*p* < 0.05); ^e^ Significant difference compared to FTE (*p* < 0.05); Nd = Not detectable.

**Table 3:** The effect of the FTE on serum ALT and hepatic MDA in liver injury induced by CCl_4_ in rats.

Groups	ALT (U/L)	MDA (nmol/g Liver)
Normal	25.49 ± 2.41	0.02 ± 0.00
CCl_4_	301.04 ^a^ ± 13.88	0.14 ^a^ ± 0.01
FTE (500 mg/kg) + CCl_4_	292.34 ± 9.38 (3.16%) ^b^	0.12 ± 0.01 (24.44%) ^b^
FTE (1000 mg/kg) + CCl_4_	206.42 ^c^ ± 18.42 (34.34%) ^b^	0.09 ^c^ ± 0.02 (48.96%) ^b^
FTE (2000 mg/kg) + CCl_4_	111.64 ^c^ ± 2.91 (68.74%) ^b^	0.04 ^c^ ± 0.00 (90.27%) ^b^
FTE (2000 mg/kg)	28.64 ^c^ ± 2.39	0.03 ^c^ ± 0.00
Silymarin (100 mg/kg) + CCl_4_	79.38 ^c^ ± 28.22 (80.43%) ^b^	0.05 ^c^ ± 0.01 (78.78%) ^b^

Values are the mean ± SEM of eight rats (in duplicate). ^a^ Significantly different from the normal control group (*p* < 0.05); ^b^ Percent inhibition of serum ALT and hepatic MDA; the percent inhibition is calculated as 100 × [(values of CCl_4_ control − values of sample)/(values of CCl_4_ control − values of normal)]; ^c^ Significantly different from the group treated with CCl_4_ alone (*p* < 0.05).
